# MicroRNA signatures in plasma and plasma exosome during window of implantation for implantation failure following in-vitro fertilization and embryo transfer

**DOI:** 10.1186/s12958-021-00855-5

**Published:** 2021-12-07

**Authors:** Hong Zeng, Yu Fu, Lang Shen, Song Quan

**Affiliations:** 1 Department of Gynecology and Obstetrics, NanFang Hospital, Southern Medical University, Guangzhou, 510515 Guangdong China; 2 Department of Reproductive Medicine Center, Foshan Maternal and Child Health Care Hospital, Southern Medical University, Foshan, 528000 Guangdong China

**Keywords:** Implantation failure, MicroRNA, Exosome, Window of implantation

## Abstract

**Background:**

MicroRNAs (miRNAs) are small, non-coding RNAs that are dysregulated in many diseases and can act as biomarkers. Although well-studied in cancer, the role of miRNAs in embryo implantation is poorly understood. Approximately 70% of embryos fail to implant following in-vitro fertilization and embryo transfer, 10% of patients experienced recurrent implantation failure. However, there are no well-established biomarkers that can predict implantation failure. Our purpose is to investigate distinct miRNA profiles in plasma and plasma exosomes during the window of implantation between patients with failed implantation and successful implantation.

**Methods:**

We select a nested case-control population of 12 patients with implantation failure or successfully clinical pregnancy using propensity score matching. RNA was extracted from plasma and plasma exosomes collected during the window of implantation (WOI). MicroRNA expression in all samples was quantified using microRNA sequencing. The intersection of differently expressed miRNAs in plasma and exosomes were further validated in the GEO dataset. Significantly altered microRNAs in both plasma and plasma exosomes were then subjected to target prediction and KEGG pathway enrichment analyses to search for key signaling pathways. WGCNA analysis was performed to identify hub miRNAs associated with implantation.

**Results:**

13 miRNAs were differentially expressed in both plasma and plasma exosomes in patients with implantation failure. Among them, miR-150-5p, miR-150-3p, miR-149-5p, and miR-146b-3p had consistent direction changes in endometrium of patients with recurrent implantation failure (RIF), miR-342-3p had consistent direction changes in blood samples of patients with RIF. Pathway enrichment analysis showed that the target genes of differentially expressed miRNAs are enriched in pathways related to embryo implantation. WGCNA analysis indicated that miR-150-5p, miR-150-3p, miR-146b-3p, and miR-342-3p are hub miRNAs.

**Conclusions:**

Implantation failure is associated with distinct miRNA profiles in plasma and plasma exosomes during WOI.

**Supplementary Information:**

The online version contains supplementary material available at 10.1186/s12958-021-00855-5.

## Background

MicroRNAs (miRNAs) are small, non-coding functional RNAs that are approximately 19–25 nucleotides in length, which mainly regulate protein expression by targeting the 3′-untranslated region (3′-UTR) of the mRNA [1]. MicroRNAs play a variety of roles in diverse diseases and can act as biomarkers [2]. Though miRNAs profiles are well studied in cancers, the role of miRNAs in embryo implantation is poorly understood. Approximately 70% of embryos fail to implant following in-vitro fertilization and embryo transfer (IVF-ET), 10% of patients even experienced recurrent implantation failure (RIF) [3]. RIF is a common and intricate complication following IVF-ET, which refers to the situation that good-quality embryos repeated fail to implant following two or more IVF cycles [4].

There are no well-established biomarkers that can predict implantation failure. Emerging literature has demonstrated that miRNAs regulate the expression of the genes involved in the establishment of the window of implantation (WOI), dysregulation of miRNAs could contribute to the implantation failure or RIF experienced by infertile patients [5–9]. Chen et.al [10] reported that the expression of hsa-miR-20b-5p, hsa-miR-155-5p, and hsa-miR-718 in endometrium was capable of predicting patients with RIF with an accuracy > 90%. It is reported that 3800 genes could be regulated by only 13 microRNAs in the endometrium of patients with RIF [11]. The above studies indicate miRNA biomarkers are promising for predicting implantation outcomes. However, endometrial biopsy is invasive and can not be performed in the embryo transfer cycle. Therefore, novel non-invasive biomarkers for the prediction of implantation failure are urgently needed. It is well studied that circulating miRNAs can act as biomarkers in various cancers [12]. However, reliable non-invasive biomarkers are not yet available for embryo implantation. Plasma miRNAs, especially exosomal microRNAs, have considerable potential as novel non-invasive biomarkers for embryo implantation [5–7, 13, 14]. Exosomes are 50-150 nm endosome-derived extracellular vesicles that are widely distributed in body fluids, such as blood, urine, ascites, and amniotic fluid [15]. Exosomes play an important role in the exchange of biological information between different cells [16]. Considering that exosomes are protected from RNase degradation, stable miRNAs can be detected in exosomes. Studies have explored circulating exosomal miRNAs as noninvasive biomarkers in some diseases [17]. The present study aims to identify the distinct miRNAs in plasma and plasma exosomes during WOI and explore hub miRNAs associated with embryo implantation.

## Methods

### Ethics approval

The study was approved by the Ethics Committee of Nanfang Hospital (accession number NFEC-2021-135). Informed consent was obtained from all the participants.

### Patients

We prospectively recruited patients who underwent routine IVF-ET at the Reproductive Medicine Center of Nanfang Hospital from October 2020 to December 2020. The inclusion criteria were: age < 40 years, with regular menstrual cycle, 18.5 kg/m^2^ ≤ BMI < 24 kg/m^2^, ≤7 mm endometrial thickness < 12 mm, infertility cause is a simple tubal factor, transferred with 1 ~ 2 good-quality embryos. The exclusion criteria were: PGD cycles, oocyte donor cycles, combined with endometriosis, PCOS, uterine abnormality, endocrine diseases, abnormal karyotypes. Blood samples were collected at WOI (LH + 7 or P + 5), patients were transferred with 1 ~ 2 good-quality embryos and followed-up for pregnancy outcomes.

### Sample size and power estimation

The “RnaSeqSampleSize” R package (version 2.2.0.) {Shilin Zhao Developer, 2021} was used to estimate the sample size for RNA-seq data. First, we estimate the dispersion distribution using the miRNA read count matrix from GSE108966. GSE108966 investigate the endometrial and blood miRNA profiles in the RIF patients using miRNA-seq technology. Only blood samples collected during the WOI were used for dispersion estimation. Because GSE108966 has two study cohorts, we estimate data dispersion of the two cohorts, respectively. The average dispersion of the two cohorts was used for the following sample size estimation and power estimation. When performing power estimation, we set the parameters as the following: phi0 = 0.24, rho = 2, k = 1, lambda0 = 50, m = 1000, f = 0.3 (phi0 denote the dispersion; rho denote the minimum fold changes for prognostic genes between two groups; k denote the ratio of sample size between two groups; lambda0 denotes the average read counts for prognostic genes; m denotes the total number of genes for testing; f denotes the false discovery rate level). The false discovery rate (FDR) was set at 0.3 due to the financial budget. Our minimum acceptable statistical power is at least 0.7.

### Plasma sample preparation and exosome isolation

Peripheral blood samples (5-6 ml per patient) were obtained from patients undergoing IVF-ET. The blood was collected into the EDTA tubes. After the blood was collected, the tubes were inverted five times, stored on ice, and processed within 30 min. Each specimen was centrifuged at 1500 g for 15 min at 4 °C to separate plasma from cells. The supernatant was then collected from each tube and transferred to new tubes (1 ml plasma per tube). The plasma (2-3 ml per patient) was stored at − 80 °C until exosome isolation. The exosome isolation was performed using the Exosome Isolation Kit from plasma (SHBIO corp. Shanghai, China, #31902–10) following the manufacturer’s instructions. 1 ml of plasma was incubated with 5 μL of thrombin at room temperature for 15 min, then centrifuged at 10,000 rpm for 5 min. The supernatant was then transferred into a sterile vessel and incubated with 250 μL of Exosome Precipitation Solution at 4 °C for 30 min, followed by centrifugation at 1500 g for 30 min. Exosome pellets were resuspended in 200 μL of PBS and stored at − 80 °C until RNA isolation.

### Transmission electronic microscopy

An aliquot of EV sample was placed on the surface of a carbon-coated copper grid for 5 min, and the extra liquid was removed. The attached EVs were negatively stained using 2% phosphotungstic acid and air-dried. EV-containing grids were observed using a transmission electron microscope (Tecnai G2 spirit Biotwin).

### Nanoparticle tracking analysis

The isolated exosomes were resuspended in 500 μl of PBS and analyzed using the NanoSight NS300 System (Malvern Instruments, UK). The movement of pellets under Brownian motion was recorded for 60 s to analyze the particle concentrations and size distribution profiles. Data were analyzed with ZetaView (Version 8.04.02).

### RNA extraction

The miRNAs were extracted from the plasma or exosomes by using the mirVana™ miRNA Isolation Kit (Cat #. AM1561, Austin TX, USA) according to the manufacturer’s protocol. The quality and distribution of miRNAs were determined using the Agilent 2100 Bioanalyzer (Agilent Technologies Santa Clara, USA).

### MicroRNA sequencing

The miRNA sequencing library was constructed using the QIAseq miRNA Library Kit (QIAGEN, German). Briefly, the total RNA of each sample was used to prepare the miRNA sequencing library as the following steps: 3′-adaptor ligation, 5′-adaptor ligation, cDNA synthesis, PCR amplification, and gel purification. After quantification with Qubit (Thermo Fisher, USA), the libraries were captured on cBOT (Illumina, USA) to be amplified in situ as clusters, and finally, they were sequenced on the Illumina HiSeq Nova (Illumina, USA) as the manufacturer’s instructions. After sequencing, the adaptor sequences were trimmed and the quality-filtered reads were harvested as clean reads. The clean reads were clustered to unique sequences and mapped to databases of the human genome, RFam, RepBase, mRNA database, miRBase using bowtie software, allowing up to one mismatch. Based on the miRNA biogenesis model, we used the miRCat software to predict novel miRNAs. Then, the clean reads of each sample were aligned to merged miRNA databases (known miRNAs from miRBase plus the newly predicted miRNAs) to calculate the miRNA expression levels. The numbers of mapped tags were defined as the raw expression levels of that miRNAs.

### Differentially expressed genes (DEG) screening

The DESeq2 (version1.32.0) R package [18] was used to screen the DEGs between the patients with failed implantation and patients with clinical pregnancy. The DEGs were selected by *p*-value < 0.1 and |log2 fold change (FC)| > 1.

### MicroRNA validation in GEO dataset

To increase the generalization and external validity of the results obtained here. We further validate the intersection of differently expressed miRNAs in plasma and exosomes using the GEO miRNA-sequencing dataset. We use the search term “implantation failure” to search gene expression datasets in Gene Expression Omnibus (GEO) database (http://www.ncbi.nlm.nih.gov/geo/). The raw data were retrieved from GEO. Datasets that met the following inclusion criteria were included: (1) gene expression profile by miRNA sequencing; (2) the tissue (endometrium or blood or both) were collected during the window of implantation; (3) patients with implantation failure and fertile control were contained in one experiment; (4) there were at least three replicates in each group. Raw counts data were downloaded from the GEO database. The Wilcoxon test was performed for between-group comparison. The level of statistical significance was set at a *p*-value < 0.05 on the two-sided test.

### MicroRNA target prediction, KEGG pathway enrichment analysis

MicroRNA target prediction and KEGG pathway enrichment analysis were performed in mirPath v.3 (http://snf-515788.vm.okeanos.grnet.gr/) [19]. The database integrates three miRNA target gene databases including TarBase, TargetScan, and microT-CDS. R software (Version4.1.0) was used for graphic visualization. In KEGG pathway enrichment analysis, cancer-related pathways were excluded in the final visualization.

### MicroRNA coexpression network construction

The coexpression network for the miRNAs was constructed by the WGCNA (version1.70–3) R package [20]. The network construction includes the following steps: (1) define the similarity matrix; (2) select the weighting coefficient, and transform the similarity matrix into an adjacency matrix; (3) transform the adjacency matrix into a topological overlap matrix (TOM); (4) perform hierarchical clustering for TOM-based dissimilarity (dissTOM) to obtain the hierarchical clustering tree; (5) use the dynamic tree cut method to identify the modules from the hierarchical clustering tree and (6) calculate the module eigengene (ME) of each module. ME represents the overall expression level of the module. The Pearson correlation coefficients between the MEs of all modules were calculated, and the 1-Pearson correlation coefficient was defined as the average distance between the MEs of all modules. The average-linkage hierarchical clustering method based on a minimum size (gene group) of 30 was employed to cluster the MEs of all modules, and the modules with high similarity were merged to obtain the coexpression network. The Cytoscape software (version 3.8.2) was used for graphic visualization.

### Identification of clinically significant modules

The correlations between the modules and clinical traits were used to estimate the module-trait associations. The Pearson correlation coefficients between the MEs of each module and each clinical trait were calculated, which permitted the identification of modules that were potentially related to the trait (*P* < 0.1).

### Identification of hub miRNAs

Hub genes are of functional importance. Hub miRNAs screening requires the determination of module membership (MM) to measure the correlation between the gene and a given module. For each gene, we define the MM by the correlation between the gene expression profile and the ME of a given module. Highly connected intramodular hub genes tend to have high MM values to the respective module. In short, the larger the MM value of the gene, the higher the correlation between the gene and a given module. In this study, we used the networkScreening function based on GS (representing the correlation between the gene and a given clinical trait) and MM (representing the correlation between the gene and a given module) in the WGCNA package to directly identify hub genes. We employed a p-weighted cutoff < 0.01 to obtain hub miRNAs.

### Statistical analysis

Continuous variables are presented as mean ± standard deviation (SD) for normally distributed data, or as median and interquartile range. Normally distributed data were compared using the Student’s t-test and non-normally distributed data using the Mann-Whitney U test. A *p*-value less than 0.05 was considered to be statistically significant. All analyses were performed using R software (version 4.1.0).

## Results

### Clinical characteristics of the patients

Plasma and plasma exosomes were collected and isolated from patients with failed implantation or successfully clinical pregnancy. To estimate the sample size needed to achieve the desired statistical power within the financial budget. We applied the “RnaSeqSampleSize” package {Shilin Zhao Developer, 2021} to estimate the sample size. The average dispersion of miRNA read count matrix from GSE108966 is 0.24. We then performed the power estimation with the dispersion parameter set as 0.24, minimum FC set as 2, FRD set as 0.3. As shown in the power curve (Supplementary Fig. 1), when the sample size in each group is 6, the power is 0.71. Our minimum acceptable statistical power is at least 0.7. The cost of miR-seq is within budget when the sample size of each group is 6. Therefore, in the miR-seq experiment, 6 patients with failed implantation were matched with 6 patients with successful clinical pregnancy by propensity score matching. Variables included in the PS model were age, endometrial thickness, number of transferred embryos, embryo stage. The clinical characteristics between the implantation failure group and the clinically pregnant group are summarized in Table 1. According to the morphologic criteria, only embryos with good quality were included. For cleavage embryos, the blastomeres were in equal size and no cytoplasmic fragments were observed, while, the blastocysts included were fully expanded with a clear structure of the inner cell mass and trophectoderm. Age, endometrial thickness, number of transferred embryos, embryo stage were not significantly different between the two groups.

**Table 1:** Clincial charateristics of patients included in the study

	CP group (*n* = 6)	IF group (*n* = 6)	*P*-value
Age (year)	32.0 ± 3.22	33.2 ± 3.60	0.568
Endometrial thickness (mm)	21.2 ± 1.58	22.7 ± 1.23	0.868
No of transferred embryos			1.0
1	3 (50%)	3 (50%)	
2	3 (50%)	3 (50%)	
Type of transferred embryos			1.0
Cleavage	4 (66.7%)	4 (66.7%)	
Morula/blastocyst	2 (33.3%)	2 (33.3%)	

*CP* Clinical pregnancy, *IF* Implantation failure

### Isolated exosomes characterization

**Fig. 1:**
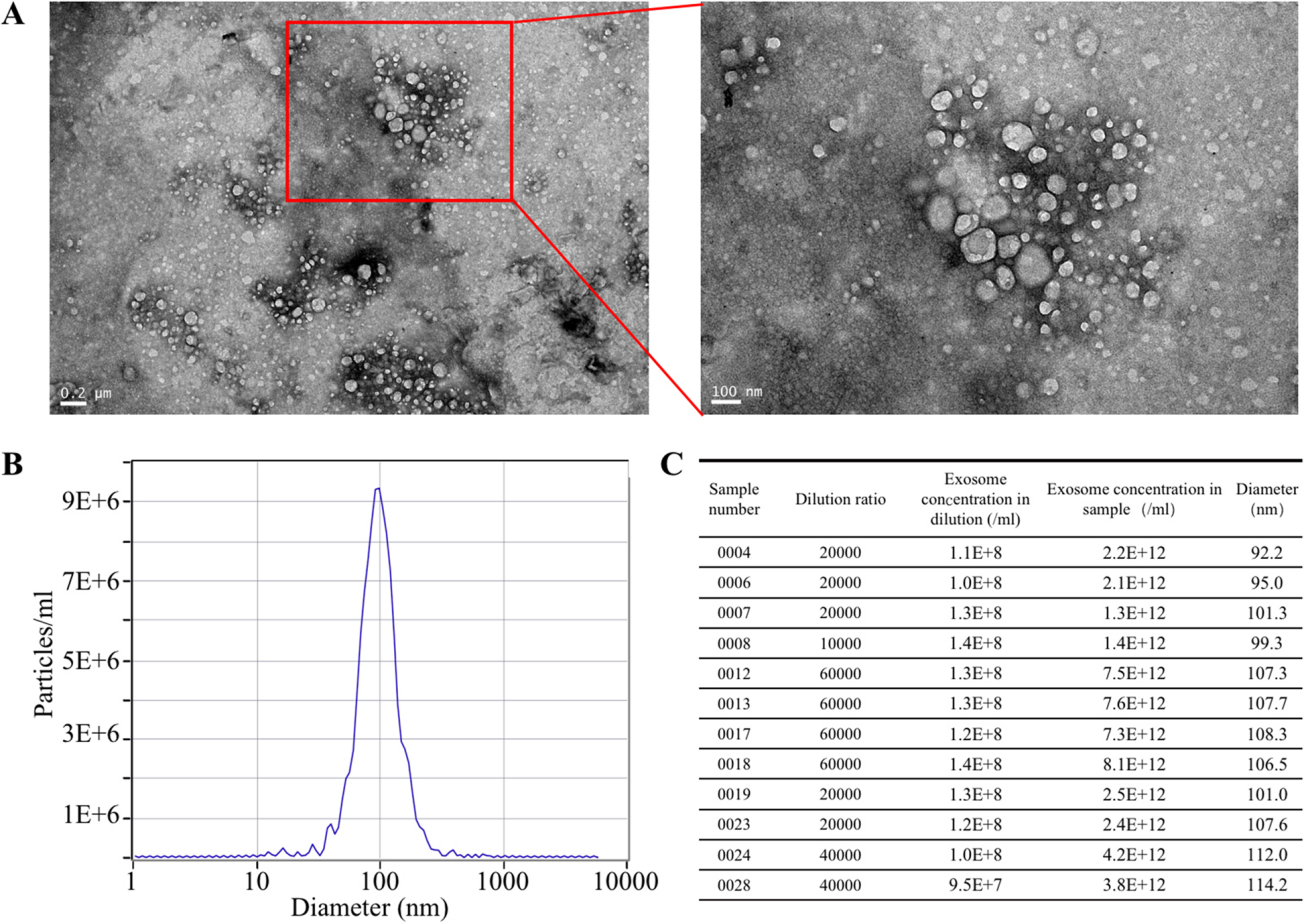
Identification of isolated plasma exosomes. **A** Representative of transmission electron microscope images of isolated exosomes. **B** Representative of NanoSight analysis for plasma exosomes. The number of particles reaches a peak in particle diameter of 100 nm. **C** Summary of exosome concentration and exosome diameter of each sample. The concentration of exosomes in the sample solution ranges from 1 ~ 8 × 10^12^ particles/ml

TEM and NTA were used to determine whether the exosomes were successfully isolated. TEM showed that isolated exosomes had a round or oval shape (Fig. 1A). NanoSight analysis demonstrated that the diameter distribution of exosomes ranged from approximately 50 nm to 150 nm in diameter (Fig. 1B, C). Nanoparticle calculations based on the NTA experiments revealed that the concentration of exosomes in the sample solution was 1 ~ 8 × 10^12^ particles/ml (Fig. 1D).

### MicroRNA profiling in plasma and plasma exosomes at WOI

**Fig. 2:**
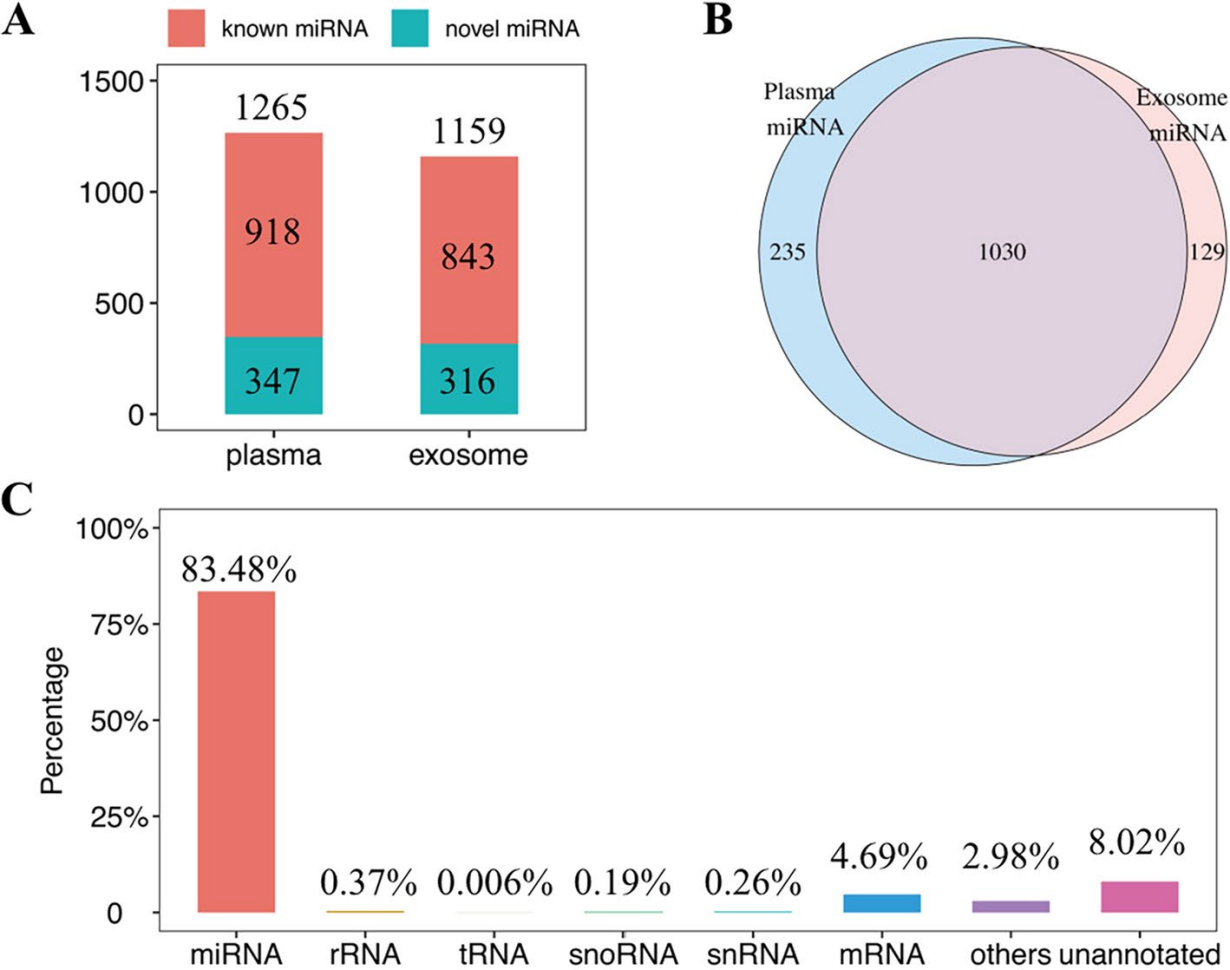
MicroRNA profile of plasma and plasma exosomes at WOI. **A** The proportion of known miRNAs and novel miRNAs detected in plasma and exosomes. **B** The intersection of 1030 miRNAs detected in plasma and plasma exosomes. **C** The proportion of small RNA types

We performed miRNA sequencing in the plasma and plasma exosomes samples derived from patients with failed implantation and successfully clinical pregnancy to identify the differential miRNAs. 1265 and 1159 miRNAs were detected in the plasma and plasma exosomes, respectively. Among all the detected miRNAs, the numbers of novel predicted miRNAs in plasma and plasma exosomes were 347 and 316, respectively (Fig. 2A). Venn plot showed that 1030 miRNAs detected in plasma exosomes were also present in plasma (Fig. 2B). MiRNAs accounted for 83.48% of all the small RNAs species in plasma and plasma exosomes (Fig. 2C).

### Identification of differentially expressed miRNAs in plasma or plasma exosomes from patients with different pregnancy outcomes

**Fig. 3:**
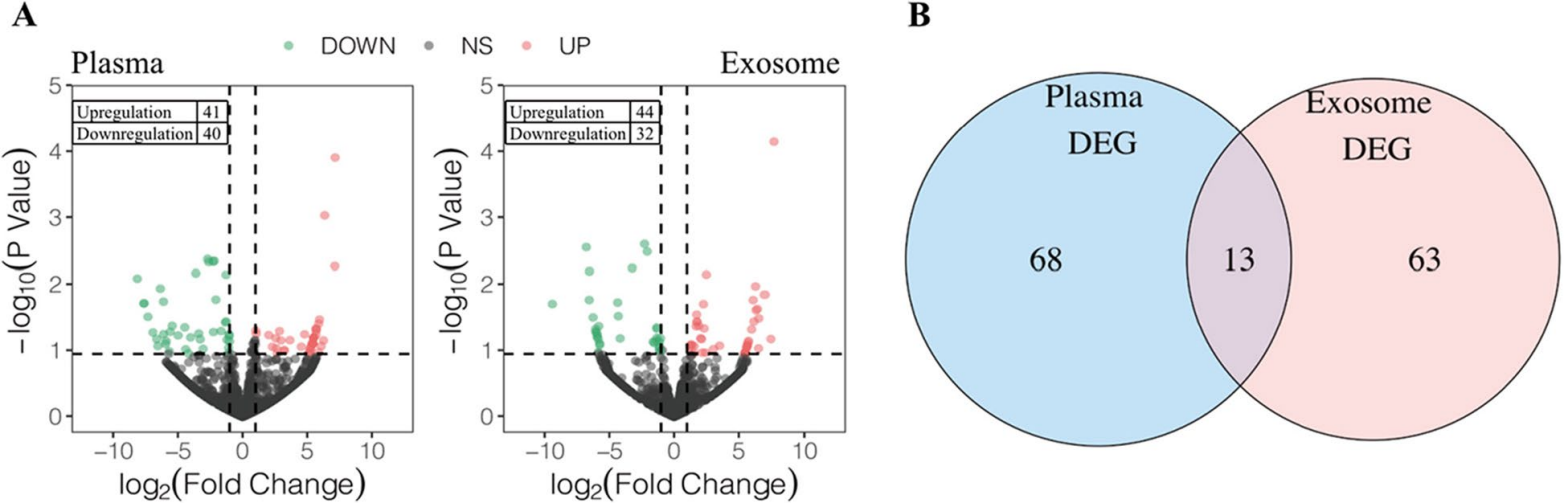
Identification of differentially expressed miRNAs. **A** Volcano plot showing the 81 differentially expressed miRNAs (41 up-regulated, 40 down-regulated) between the IF group and CP group in plasma and 76 differentially expressed miRNAs (44 up-regulated, 32 down-regulated) in the plasma exosomes, respectively. **B** Venn plot showing the intersection of 13 differentially expressed miRNAs in plasma and plasma exosomes

A total of 81 differentially expressed miRNAs were identified in the plasma, with 41 miRNAs up-regulated and 40 miRNAs down-regulated (Fig. 3A, left). A total of 76 differentially expressed miRNAs were identified in the plasma exosomes, with 44 miRNAs up-regulated and 32 miRNAs down-regulated (Fig. 3A, right). 13 miRNAs were differentially expressed in both plasma and plasma exosomes (Fig. 3B). Among the 13 miRNAs, one is a novel miRNA. The other 12 are known miRNAs. Three of the 12 known miRNAs (miR-6767-5p, miR-149-5p, miR-4433b-5p) are upregulated in both plasma and plasma exosomes, seven of the 12 known miRNAs (miR-4511, miR-124-3p, miR-146b-3p, miR-150-5p, miR-150-3p, miR-342-3p, miR-874-3p) are downregulated in both plasma and plasma exosomes, while miR-100-5p and miR-125b-5p have the opposite direction changes in plasma and plasma exosomes. The result of DEGs in plasma was shown in Supplementary Table 1. The result of DEGs in plasma exosomes was shown in Supplementary Table 2.

### Validation in GEO dataset

**Fig. 4:**
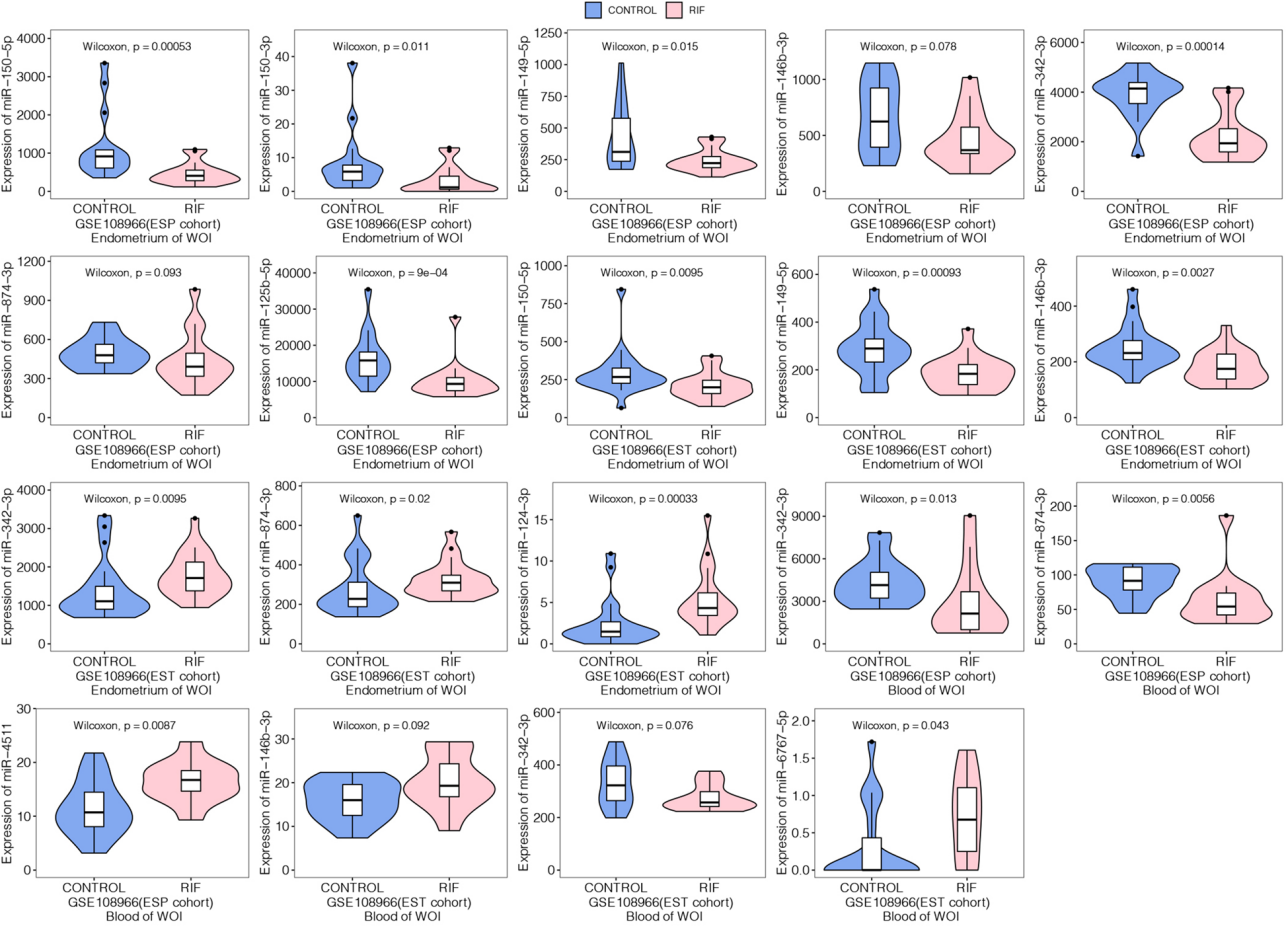
Violin plot showing the validation of differently expressed miRNAs in the GEO108966 dataset

12 known miRNAs (miR-6767-5p, miR-4511, miR-149-5p, miR-124-3p, miR-146b-3p, miR-100-5p, miR150-5p, miR-342-3p, miR-150-3p, miR-4433b-5p, miR-874-3p, miR-125b-5p) of the intersection of differently expressed miRNAs in plasma and plasma exosomes were further validated in the GSE108966 dataset. GSE108966 dataset includes two cohorts that investigated distinct miRNA profiles from the endometrium and whole blood in RIF patients and fertile patients. We extracted raw counts data of the endometrial tissue and blood during WOI from 60 patients and 71 patients in cohort ESP and cohort EST, respectively. The counts’ data were normalized by the DESeq2 R package. According to the expression profile analysis in the ESP cohort of the GSE108966 dataset, miR-150-5p, miR-150-3p, miR-149-5p, miR-342-3p, and miR-125b-5p showed significantly lower expression in the endometrium of the RIF group compared with the control group (*P* < 0.05). MiR-342-3p and miR-874-3p showed significantly lower expression, while miR-4511 showed significantly higher expression in the blood of the RIF group compared with the control group (*P* < 0.05). On the other hand, we found a trend for lower miR-146b-3p and miR-874-3p in the endometrium of the RIF group, although no statistical differences were reached (both 0.05 < *P* < 0.1). In cohort EST of GSE108966 dataset, the expression of miR-150-5p, miR-149-5p and miR-146b-3p were also significantly lower, while miR-342-3p, miR-874-3p, miR-124-3p were significantly higher in the endometrium of RIF patients (*P* < 0.05). MiR-342-3p showed significantly lower expression, while miR-146b-3p and miR-6767-5p showed significantly higher expression in the blood of the RIF group compared with the control group (*P* < 0.05). The corresponding violin plots were shown in Fig. 4. In general, the regulation directions of miR-150-5p, miR-150-3p, miR-149-5p, and miR-146b-3p in the endometrium sample of the GSE108966 dataset were all accordant with our results in plasma and plasma exosomes. The regulation direction of miR-342-3p in blood samples of the GSE108966 dataset was accordant with our results in plasma and plasma exosomes.

### KEGG pathway enrichment analysis

**Fig. 5:**
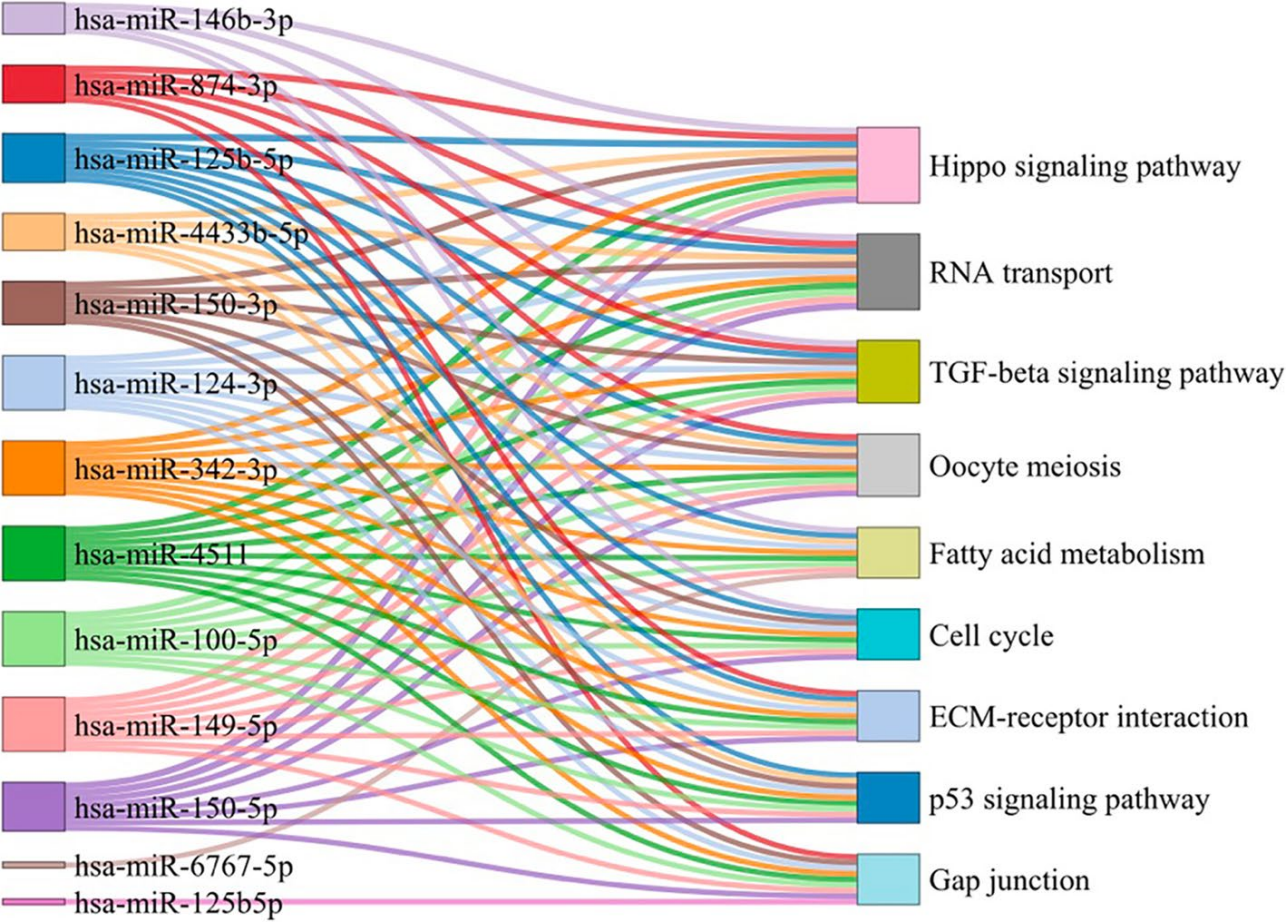
Sanke plot showing the top enriched KEGG pathways and the corresponding miRNAs

The KEGG signaling pathways associated with these differentially expressed miRNAs in plasma and exosomes were enriched in the pathways of Hippo signaling pathway, RNA transport, TGF-beta signaling pathway, Oocyte meiosis, Fatty acid metabolism, Cell cycle, ECM-receptor interaction, p53 signaling pathway, and Gap junction (Fig. 5). All of these pathways are related to embryo implantation. The result of KEGG enrichment analysis was shown in Supplementary Table 3.

### Weighting coefficient β selection

**Fig. 6:**
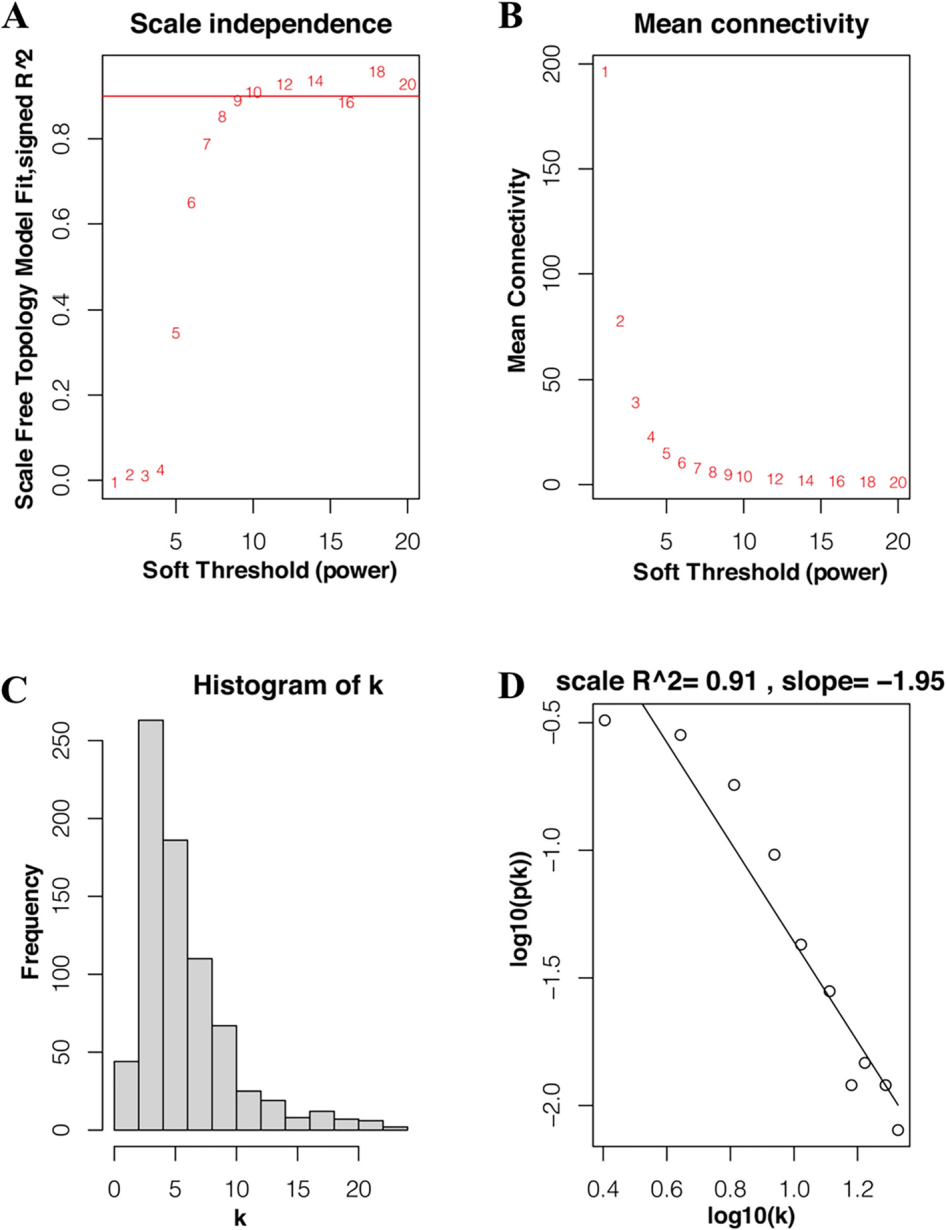
Weighting coefficient β selection. **A** Analysis of network topology for various soft-thresholding powers (weighting coefficient β). The x-axis represents different soft-thresholding powers. The y-axis represents the correlation coefficient between log (k) and log [P(k)]. The red line indicates a correlation coefficient of 0.9. **B** Average network connectivity under different weighting coefficients. **C** Distribution of nodes with the degree of connection, k. **D** Correlation of log (k) and log [P(k)]

Considering the correlation coefficient and mean connectivity, it was reasonable to choose β = 9 to construct the co-expression network. When choosing β = 9, the corresponding correlation coefficient is 0.889 the mean connectivity is 4.71 (Fig. 6A, B). By analyzing Fig. 6C and D, we can conclude that when β = 9, the co-expression network is a scale-free network.

### Identification of MicroRNA coexpression modules correlated with implantation

**Fig. 7:**
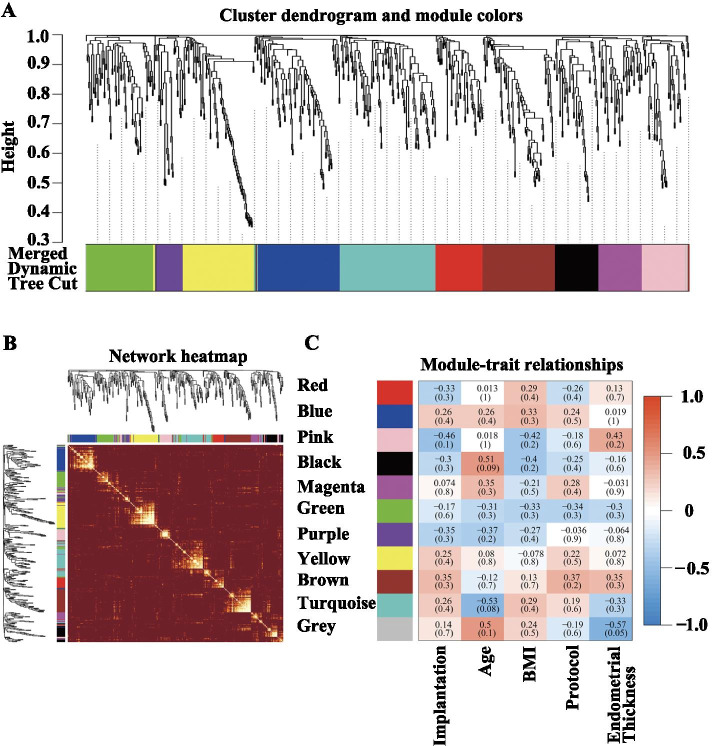
Cluster dendrogram, network heatmap, and relationships between modules and clinical traits. **A** Clustering dendrograms of all miRNAs, with dissimilarity based on topological overlap, together with assigned module colors. **B** Heatmap plot for visualizing the gene network. Darker red indicates low overlap, and light color indicates greater overlap. The module assignment gene and dendrogram are also shown along the top and left sides. **C** Module-trait associations. Each column corresponds to a trait, and each row corresponds to a ME. The number in the rectangle is the correlation coefficient, and the number in brackets is the corresponding *p*-value. The table is color-coded by correlation based on the color legend

**Fig. 8:**
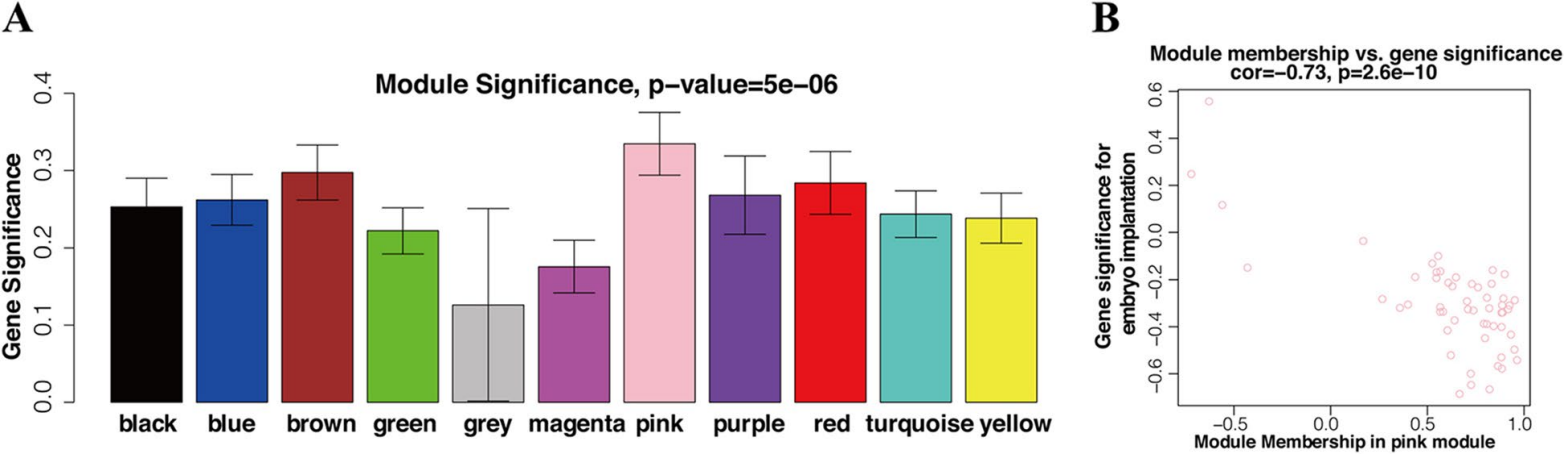
Module and eigengene network plot. **A** Bar plots of mean GS across modules. The higher the mean GS in a module is, the more significantly associated with implantation the module will be. **B** Scatterplots of GS for implantation (y-axis) versus MM (x-axis) in the pink module

A hierarchical clustering tree was obtained by conducting hierarchical clustering for dissTOM (Fig. 7A). According to the dynamic tree cut method, the final number of obtained miRNA coexpression modules was 10 (grey denotes the genes that were not divided into any cluster) (Fig. 7A). Figure 7B showed the relations of module genes. The Pearson correlation coefficients for the MEs of all modules and the clinical information were calculated to identify which modules were related to the clinical traits. Correlation analysis between modules and clinical traits indicated that the pink module was potentially negatively associated with implantation (*R* = − 0.46, *P* = 0.1) (Fig. 7C). Highly co-expressed genes in the same module have potential biological significance. Therefore, the pink module was treated as an implantation module in subsequent analyses. To ensure the reliability of the identification results for the implantation module, the module was identified again by calculating the mean absolute GS value of the genes for implantation in each module. The mean absolute GS values for implantation were largest for the pink module; that is, the pink module exhibited the highest correlation with implantation failure (Fig. 8A). The MM value for each gene was calculated to identify hub genes in the module. Correlation analysis between GS for embryo implantation and MM for genes in each module was performed to test whether the MM value was closely related to implantation failure. The results showed that the correlation coefficient between GS for embryo implantation and MM was highest in the pink module (correlation coefficient = − 0.73, *p*-value = 2.6e-10); that is, the pink module exhibited the greatest negative correlation with embryo implantation, consistent with the above conclusions. Therefore, the identification of hub genes was performed within the pink module.

### Identification of hub miRNAs of implantation failure

**Fig. 9:**
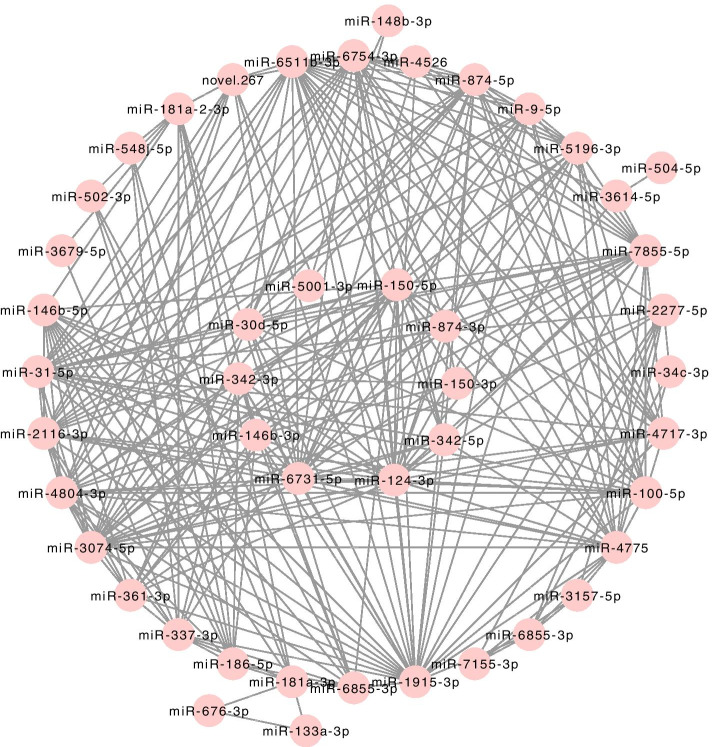
Network plot of genes in pink module and hub miRNAs. MiRNAs in the inner circle denotes the hub miRNAs

It has been demonstrated that the MM value can reflect the correlation between genes and embryo implantation. The larger the MM value of a gene is, the higher the correlation between the gene and a given module, and the more relevant the gene is to embryo implantation. In this study, we identified hub genes using the ‘networkScreening’ function based on GS for embryo implantation and MM. Each gene has its own p-weighted value, and we employed a p-weighted cutoff < 0.01 to obtain 10 genes that were highly correlated with embryo implantation. Hub gene screening was required to meet two conditions. The first was that the hub genes were in the module (pink module) that were significantly related to embryo implantation. The second was that the hub genes met the screening criteria (p-weighted < 0.01) of the ‘networkScreening’ function of the WGCNA method. Once a gene met these two screening conditions at the same time, it was defined as a hub gene. As a result, a total of 10 hub genes (miR-150-5p, miR-874-3p, miR-150-3p, miR-342-5p, miR-124-3p, miR-6731-5p, miR-146b-3p, miR-342-4p, miR-30d-5p, miR-5001-3p) were screened out in the pink module, hub miRNAs was shown in Fig. 9 located in the inner circle. Among the hub miRNAs, four miRNAs (miR-150-5p, miR-150-3p, miR-146b-3p, and miR-342-3p) were also significantly DEGs in both plasma and exosomes in our study and they were also significantly regulated miRNAs validated in GSE108966. The Result of hub genes in the pink module was shown in Supplementary Table 4.

## Discussion

Embryo implantation rate following IVF-ET hit a bottleneck with approximately 70% of embryos failing to implant, 10% of patients even experienced recurrent implantation failure. Implantation is a multifactorial process that involves delicate coordination between the embryo and receptive endometrium. Accumulating evidence has demonstrated that the endometrial factor serves as the main factor for implantation, especially when morphological good-quality embryos are selected to transfer. It is well-known that the window of implantation (WOI) is a short time after ovulation which allows embryos to implant. MicroRNA dysregulation involved in the establishment of WOI could contribute to implantation failure. Though, it is reported that the expression of endometrial miRNA profiles was capable of predicting patients with RIF with high accuracy [10]. However, reliable non-invasive biomarkers are not yet available for embryo implantation outcomes. Endometrial cells may secrete and transport miRNAs to the tissue site of action by way of the blood, and studies suggest that endometrial expression levels of at least some miRNAs are reflected in the blood [21, 22]. Plasma miRNAs, especially plasma exosomal microRNAs, have considerable potential as novel non-invasive biomarkers for embryo implantation [5–7, 13, 14]. In the present study, we identified the circulating microRNA signature and hub miRNAs by miRNA sequencing of plasma and plasma exosome samples from patients with implantation failure and patients with successful clinical pregnancies. Our study found 13 differentially expressed miRNAs in both plasma samples and plasma exosome samples between patients with implantation failure and patients with successful clinical pregnancy. To the best of our knowledge, the use of miRNA sequencing to detect the circulating miRNAs profiles in plasma and plasma exosomes simultaneously as potential biomarkers for implantation failure has not yet been reported. Intersection miRNAs of differentially expressed miRNAs in both plasma and plasma exosomes indicates a more stable and reliable result. Moreover, we validated these miRNAs further in the GEO dataset. Among these miRNAs, miR-150-5p, miR-150-3p, miR-149-5p, and miR-146b-3p had accordant direction changes in endometrium of patients with recurrent implantation failure (RIF), miR-342-3p had accordant direction changes in blood samples of patients with RIF. Furthermore, WGCNA analysis indicated that miR-150-5p, miR-150-3p, miR-146b-3p, and miR-342-3p are hub miRNAs, while hub genes are usually of functional importance. Pathway enrichment analysis showed that the target genes of the differentially expressed miRNAs are enriched in pathways related to implantation. Collectively, our study indicates that miR-150-5p, miR-150-3p, miR-146b-3p, and miR-342-3p are potential biomarkers associated with embryo implantation.

The above miRNAs are reported to be related to embryo implantation in previous studies. It is reported that MiR-146 regulates trophoblast migration and invasion during early pregnancy [23]. miR-149 can regulate endometrial receptivity and decidualization for trophoblast implantation [24]. Polymorphisms of miR-146a C > G and miR-149 T > C is associated with susceptibility to idiopathic recurrent pregnancy loss and RIF [11, 25]. Zeng et.al reported that miR-150-5p mediates trophoblasts migration and angiogenesis in-vitro [26]. Broi’s miRNA-seq data showed that miR-150-5p was significantly decreased in the endometrium of patients with endometriosis compared to fertile control [8]. Part et.al reported that miR-150G > A polymorphism was associated with RPL [27]. Researchers found that miR-342-3p can regulate trophoblasts proliferation, migration, and invasion [28, 29]. Besides, miR-125b regulates endometrial receptivity, mediate embryo-maternal interactions [30, 31]. Di Pietro et.al reported that miR-124-3p is a new potential molecular marker of endometrial receptivity upregulated in endometrium and serum from women affected by Chronic Endometritis [22]. However, the role of miR-6767-5p, miR-4511, miR-874-5p or miR-4433b on embryo implantation has not been reported.

Though the differentially expressed miRNAs are validated in the GEO dataset. MiR-100-5p, miR-6767-5p, miR-4511, miR-124-3p, miR-125b-5p, miR-4433b-5p, and miR-874-3p exhibited opposite directional changes between our results and GEO dataset. There are the following explanations for the discrepancies between the results from our study and the GEO dataset: (1) different sequencing platform and data analysis method; (2) GSE108966 dataset includes two cohort patients, ESP cohort was conducted in Estonia, while EST cohort was conducted in Spain. Both endometrium samples and blood samples were included in this study. However, blood samples collected were whole blood samples in the EST cohort, while buffy coats (leukocytes and platelets) were collected in the ESP cohort. First, the genetic background may affect miRNA profiles due to different ethnicities. Second, miRNAs profiles are distinct between blood samples and endometrium samples. Third, miRNA profiles are different between whole blood, plasma, and plasma exosomes.

Hub genes are of functional importance. Among the ten hub miRNAs, miR-150-5p, miR-150-3p, miR-342-5p, miR-146-3p, miR-124-3p, miR-342-5p, and miR-874-3p were also differentially dysregulated in both plasma and plasma exosomes, these miRNAs are reported to be related with embryo implantation [11, 22–26, 28–31]. Some of the other hub miRNAs have also been reported to be associated with embryo implantation failure. Evidence show that miR-30d-5p plays important role in embryo implantation [32–35]. Human endometrium can secrete miR-30d which is further taken up by the pre-implantation embryo [33]. MiR-30d knock-out mice induced a significant downregulation of endometrial receptivity markers and had poorer implantation rates than wild-type embryos [32]. The expression of miR-30d-5p was significantly decreased in the endometrium of patients with RIF during WOI [35]. While miR-6731-5p and miR-5001-3p have not been reported to be associated with embryo implantation, so far.

Finally, the following limitations of our study should be emphasized. The case-control nature of our study does not allow us to derive a causal relationship between circulating miRNAs and embryo implantation outcomes. However, the recognition of potential new biomarkers associated with embryo implantation is also relevant from a clinical and public reproductive perspective. Further evaluation in larger prospective trials is warranted to assess the potential diagnostic or prognostic value of these miRNAs.

## Conclusions

Implantation failure is associated with distinct miRNA profiles in plasma and plasma exosomes during WOI.

## Supplementary Information

**Additional file 1: Supplementary Figure 1.** The power curve for sample size and power estimation.

**Additional file 2: Supplementary Table 1.** Differentially expressed miRNAs in plasma between the IF group and CP group.

**Additional file 3: Supplementary Table 2.** Differentially expressed miRNAs in plasma exosomes between the IF group and CP group.

**Additional file 4: Supplementary Table 3.** KEGG pathway enrichment analysis of target genes of the 12 known differently expressed miRNAs.

**Additional file 5: Supplementary Table 4.** Result of hub genes in pink module.

## Data Availability

The raw data and data analysis source codes are available from the corresponding author on reasonable request.
