# Supplementary figures and images for: MicroRNA signatures in plasma and plasma exosome during window of implantation for implantation failure following in-vitro fertilization and embryo transfer
Source: Reprod Biol Endocrinol. 2021 Dec 7;19:180. doi: 10.1186/s12958-021-00855-5 (PMC8650536; doi:10.1186/s12958-021-00855-5)

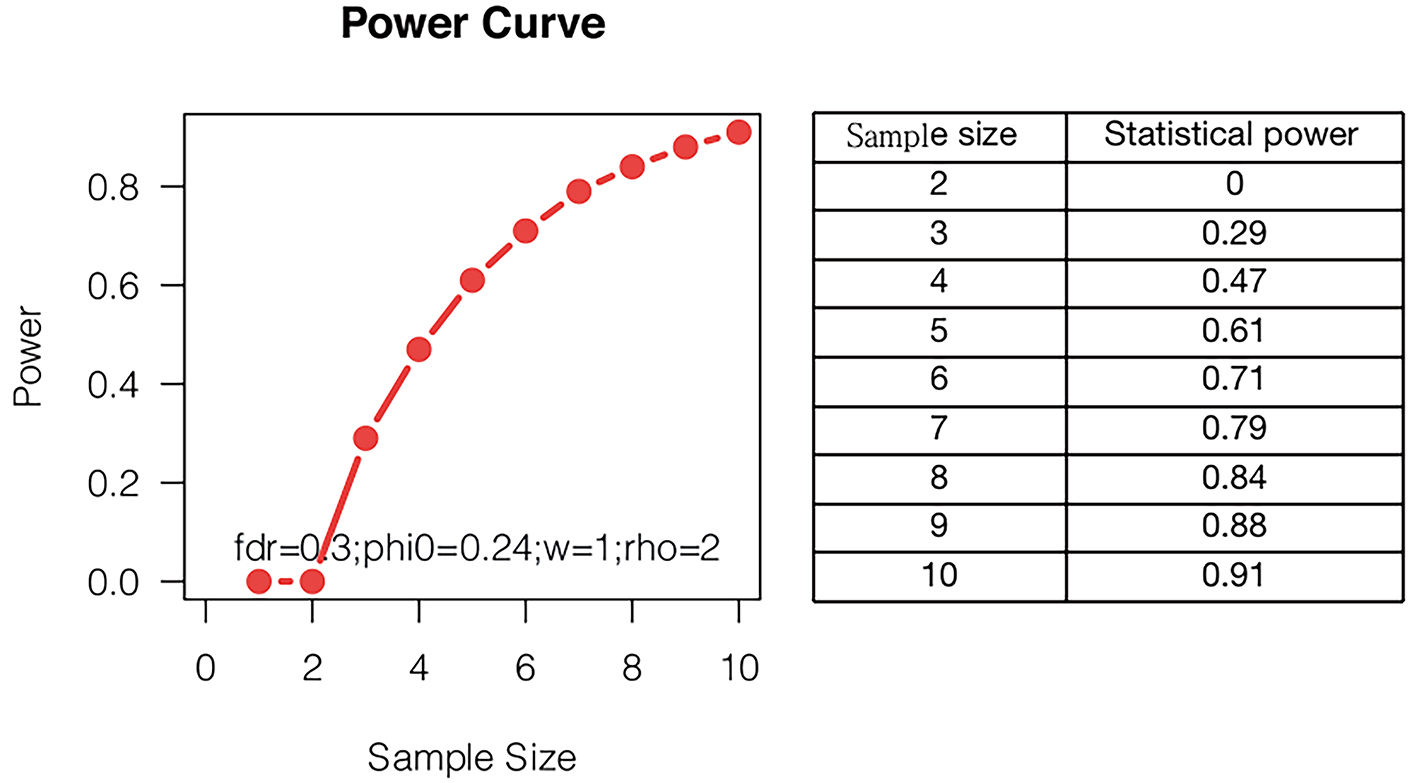

Supplement: Supplementary file 1 — Additional file 1: Supplementary Figure 1. The power curve for sample size and power estimation. [file 12958_2021_855_MOESM1_ESM.jpg]
